# The application of corneal biomechanical interocular asymmetry for the diagnosis of keratoconus and subclinical keratoconus

**DOI:** 10.3389/fbioe.2023.1266940

**Published:** 2023-10-06

**Authors:** Ruilan Dong, Yan Liu, Yu Zhang, Yueguo Chen

**Affiliations:** ^1^ Department of Ophthalmology, Peking University Third Hospital, Beijing, China; ^2^ Beijing Key Laboratory of Restoration of Damaged Ocular Nerve, Peking University Third Hospital, Beijing, China; ^3^ Peking University Institute of Laser Medicine, Beijing, China

**Keywords:** corneal biomechanics, interocular asymmetry, keratoconus, subclinical keratoconus, diagnostic model, artificial intelligence

## Abstract

**Purpose:** To evaluate the interocular consistency of biomechanical properties in normal, keratoconus (KC) and subclinical keratoconus (SKC) populations and explore the application of interocular asymmetry values in KC and SKC diagnoses.

**Methods:** This was a retrospective chart-review study of 331 ametropic subjects (control group) and 207 KC patients (KC group, including 94 SKC patients). Interocular consistency was evaluated using the intraclass correlation coefficient (ICC). Interocular asymmetry was compared between the control and KC groups and its correlation with disease severity was analyzed. Three logistic models were constructed using biomechanical monocular parameters and interocular asymmetry values. The diagnostic ability of interocular asymmetry values and the newly established models were evaluated using receiver operating characteristic curves and calibration curves. Net reclassification improvement (NRI) and integrated discrimination improvement (IDI) were also estimated.

**Results:** The interocular consistency significantly decreased and the interocular asymmetry values increased in KC patients compared with those in control individuals. In addition, the interocular asymmetry values increased with respect to the severity of KC. The binocular assisted biomechanical index (BaBI) had an area under the curve (AUC) of 0.998 (97.8% sensitivity, 99.2% specificity; cutoff 0.401), which was statistically higher than that of the Corvis biomechanical index [CBI; AUC = 0.935, *p* < 0.001 (DeLong’s test), 85.6% sensitivity]. The optimized cutoff of 0.163 provided an AUC of 0.996 for SKC with 97.8% sensitivity, which was higher than that of CBI [AUC = 0.925, *p* < 0.001 (DeLong’s test), 82.8% sensitivity].

**Conclusion:** Biomechanical interocular asymmetry values can reduce the false-negative rate and improve the performance in KC and SKC diagnoses.

## Introduction

Detection of keratoconus (KC) and especially subclinical keratoconus (SKC) plays a crucial role in preoperative screening of refractive surgeries, considering that 88% of postsurgery corneal ectasia was attributed to keratoconic eyes that failed to be ascertained before surgery (Rand et al., 2003). With the assistance of artificial intelligence (AI), the diagnostic ability of KC has indeed improved greatly in this decade (Vinciguerra et al., 2016; Ambrósio et al., 2017; Lopes et al., 2018). However, the established AI diagnostic models seem to have stepped into a bottleneck period with high but unsatisfactory sensitivity and specificity (Cao et al., 2022). There is still a need to mine more characteristic features of the disease and refine the current diagnostic models.

Corneal morphology has been indicated to have good symmetry in normal individuals in terms of keratometry, pachymetry and elevation (Durr et al., 2015; Xu et al., 2021). Keratoconus is regarded as a binocularly affected but generally asymmetrically developed disease (Gomes et al., 2015). Some research found that the interocular asymmetry of keratoconic eyes is not only greater than that of normal eyes but also increases with the severity of the worse eye (Naderan et al., 2017; Eppig et al., 2018). Then, several studies managed to construct diagnostic models for KC and very early keratoconus (VEKS) or keratoconus suspect (KCS) using the interocular asymmetry in corneal morphology, which presented the inspiring potential of morphological interocular asymmetry to assist the diagnosis of KC and SKC (Saad et al., 2014; Galletti et al., 2015a; Mehlan et al., 2022).

Furthermore, studies have confirmed that the reduction in biomechanical stability occurs prior to the change in morphology and that biomechanical weakening could be the initiating event of the disease (Scarcelli et al., 2014). However, the distribution range of most biomechanical parameters overlaps in normal individuals and SKC patients. Therefore, the effect of applying biomechanical parameters to the diagnosis of SKC is not ideal. There might be asymmetric changes in biomechanical properties first, which then lead to asymmetric morphological changes. We hypothesize that it may be beneficial to improve the sensitivity of diagnosing SKC by introducing biomechanical interocular asymmetry assessment.

In this study, we compared the interocular asymmetry between keratoconic and normal eyes and clarified the normal range of interocular asymmetry in biomechanical properties. In addition, we attempted to construct an asymmetry index based on binocular data and evaluate its auxiliary effect on monocular models for the diagnosis of KC and SKC.

## Materials and methods

This is a retrospective case–control study. The protocol followed the tenets of the Declaration of Helsinki and was approved by the institutional review board of Peking University Third Hospital. The records of patients with ametropia or keratoconus referred to the Peking University Institute of Laser Medicine were reviewed after informed consent was obtained.

### Study patients

The inclusion criteria for the control group were myopic candidates for refractive surgery with normal slit-lamp biomicroscopy, corrected distance visual acuity of 20/20 or better, and normal topography and tomography [defined as showing a normal (less than 1.6) Belin/Ambrósio total deviation index]. All control subjects enrolled in this study underwent FS-LASIK and remained in a stable refractive status during follow-up for at least 2 years. Their preoperative data were adopted in the following statistics. The criteria for the keratoconus group were the diagnosis of clinical ectasia in either eye without any previous ocular procedures, including corneal collagen cross-linking and intracorneal ring segment implantation. Clinical ectasia was diagnosed based on the presence of slit-lamp findings (e.g., Fleisher’s ring, Vogt’s striae, Munson’s sign or Rizutti’s sign), abnormal topography (e.g., skewed asymmetric bowtie or inferior temporal steepening) or tomography [defined as showing an abnormal (2.6 or greater) Belin/Ambrósio total deviation index]. All records were blindly re-evaluated by two experienced ophthalmologists (YGC and YZ) following the global consensus on keratoconus to confirm the diagnosis (Gomes et al., 2015). Patients with a history of ocular surgery or trauma, compound ocular diseases (e.g., glaucoma), corneal scarring, topical medication or systematic diseases with ocular presence were excluded.

The Keratoconus severity score (KSS) was used to grade the severity of keratoconus in this study. The KSS system consists of grades 0 (unaffected-normal topography), 1 (unaffected-atypical topography), 2 (suspect topography), 3 (affected-mild disease), 4 (affected-moderate disease), and 5 (affected-severe disease) (McMahon et al., 2006). Eyes with a greater KSS grade were defined as the “worse eye.” Patients with worse eye graded KSS 0–2 in the KC group were selected to further form an SKC subgroup (Ruiseñor Vázquez et al., 2014; Galletti et al., 2015b; Henriquez et al., 2020). The SKC subgroup was separately used only for validation with the aim of exploring the possibility of applying the interocular asymmetry feature to assist in distinguishing SKC (Galletti et al., 2015a).

### Examinations and parameters

All participants were asked to stop wearing contact lenses for at least 3 weeks before the examinations. Each subject underwent comprehensive ophthalmic examinations, including optometry, intraocular pressure measurement, slit-lamp biomicroscopy, corneal topography (Sirius, CSO, Italy), corneal tomography (Pentacam, Oculus, Germany) and corneal biomechanical examination (Corvis ST, Oculus, Germany). The aforementioned examinations were all conducted by adequately trained fixed technicians. Pentacam and Corvis ST images were captured automatically using ultrahigh-speed Scheimpflug cameras to avoid user dependency. Only qualified examinations marked “OK” were adopted. The parameters used for modeling are mainly sourced from Corvis ST and listed in Table 1.

**TABLE 1:** Definitions of the main abbreviations derived from Corvis ST.

Abbreviations	Definitions	Unit
Pachy	Central corneal thickness	μm
A1T	Time of reaching the first applanation	ms
A1V	Speed of the corneal apex at the first applanation	m/s
A2T	Time of reaching the second applanation	ms
A2V	Speed of the corneal apex at the second applanation	m/s
HCT	Time of undergoing the greatest degree of deformation and reaching the highest concavity	ms
PD	Distance between the two bending peaks created in the cornea at the highest concavity	mm
A1DeflAmp	Deflection amplitude of the corneal apex at the first applanation	mm
HCDeflAmp	Deflection amplitude of the corneal apex at the highest concavity	mm
A2DeflAmp	Deflection amplitude of the corneal apex at the second applanation	mm
A1DeflArea	Deflection area between the initial convex cornea and cornea at the first applanation on the analyzed horizontal sectional plane	mm^2^
HCDeflArea	Deflection area between the initial convex cornea and cornea at the highest concavity on the analyzed horizontal sectional plane	mm^2^
A2DeflArea	Deflection area between the initial convex cornea and cornea at the second applanation on the analyzed horizontal sectional plane	mm^2^
A1ΔArcL	Change in Arclength during the first applanation moment from the initial state	mm
HCΔArcL	Change in Arclength during the highest concavity moment from the initial state	mm
A2ΔArcL	Change in Arclength during the second applanation moment from the initial state	mm
MaxIR	Maximum inverse concave radius	mm^−1^
DAR2	Ratio between the central deformation and the average of the peripheral deformation determined at 2.00 mm	—
DAR1	Ratio between the central deformation and the average of the peripheral deformation determined at 1.00 mm	—
ARTh	Ambrósio relational thickness to the horizontal profile	—
bIOP	Biomechanically corrected intraocular pressure	mmHg
IR	Area under the inverse concave radius curve	mm^−1^
SP-A1	Stiffness parameter at the first applanation	—
SSI	Stress‒Strain Index	—
CBI	Corvis biomechanical index	—
TBI	Tomographic and biomechanical index	—
PRFI	Pentacam random forest index	—

### Statistical analysis

The interocular asymmetry value was defined as the absolute value of the right eye minus that of the left eye and represented with the prefix “Δ”. The normality was assessed using the Kolmogorov–Smirnov test. Continuous variables are described as the mean ± standard deviation, while categorical variables are expressed as number and percentages. The inter group comparisons of age and gender ratios were conducted using independent sample t-tests and chi-square tests, respectively. The intraclass correlation coefficient (ICC) was adopted to evaluate interocular consistency. The interocular asymmetry values between the control group and the KC group were compared using the Mann–Whitney *U* test, while comparisons among patients with different severities of keratoconus were conducted using the Kruskal–Wallis test.

**FIGURE 1:**
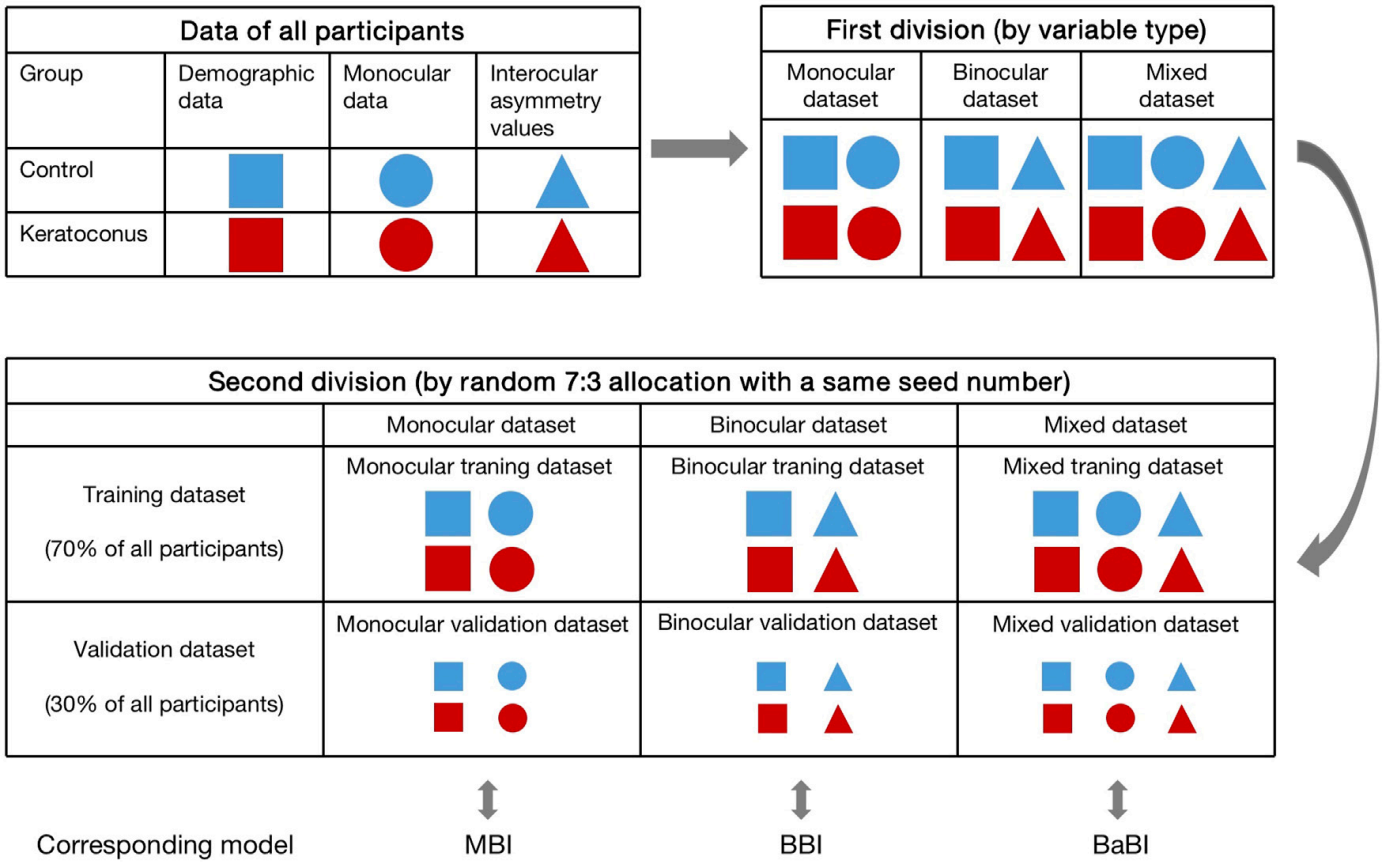
The process of dividing the datasets and the composition of each dataset. MBI, monocular biomechanical index; BBI, binocular biomechanical index; BaBI, binocular assisted biomechanical index.

To compare the diagnostic performance of interocular asymmetry values and monocular parameters, the data of all participants were categorized into three datasets according to variable type before modeling. The monocular dataset consisted of demographic data and right eye data, while the binocular dataset consisted of demographic data and interocular asymmetry values. The demographic data, right eye data and interocular asymmetry values jointly made up the mixed dataset. Six datasets (3 training datasets and 3 validation datasets) with two groups each were compiled by random 7:3 allocation of control subjects and patients with keratoconus. A same seed number was adopted in the divisions of training datasets and validation datasets to ensure consistency (Figure 1).

In the training datasets, variables were preliminarily screened by Lasso regression and then subjected to logistic regression (full model) to determine the B constants. The monocular biomechanical index (MBI) and binocular biomechanical index (BBI) were established using the monocular training dataset and binocular training dataset, respectively. The combined model, the binocular assisted biomechanical index (BaBI), was constructed by the mixed training dataset (Figure 1). Receiver operating characteristic (ROC) curves and calibration curves were drawn to assess the diagnostic ability of the biomechanical interocular asymmetry values and the logistic models. Optimal cutoff values of the newly constructed models were obtained from the ROC curves as those closest to the perfect classification point. Subsequently, the models were independently validated using corresponding validation datasets to exclude overfitting. The area under the ROC curve (AUC) of the Corvis biomechanical index (CBI), Tomographic and biomechanical index (TBI), Pentacam random forest index (PRFI) and the newly constructed models was compared using Delong’s test. Net reclassification improvement (NRI) and integrated discrimination improvement (IDI) were also estimated to evaluate the additive value of interocular asymmetry values in diagnosing KC. The nomogram was drawn for the convenience of clinical application.

The SKC subgroup was used for independent validation. The ROC curve was plotted to compare the performance of the obtained models in SKC diagnosis, and the additional value to distinguish SKC from normal provided by interocular asymmetry values was evaluated using NRI and IDI analysis.

Statistical analysis was conducted using SPSS 24.0 (IBM Corporation, Armonk, NY, United States) and R software 4.2.2 (R Foundation for Statistical Computing, Vienna, Austria). A *p*-value of less than 0.05 was regarded as statistically significant.

## Results

The composition and demographic characteristics of the analyzed datasets are shown in Table 2. A total of 331 myopia patients in the control group and 207 patients in the KC group were recruited in this study. The mean ages were 28.01 ± 7.16 and 25.17 ± 6.01 in the two groups, respectively (*p* < 0.001). There were more males in the KC group than in the control group (70.0% vs. 38.9%, *p* < 0.001). The SKC subgroup consisted of 94 patients with an average age of 24.68 ± 5.83 (*p* < 0.001, compared with the control group), of whom 67.0% were male.

**TABLE 2:** The composition and demographic characteristics of the training and validation datasets.

Demographic characteristics	Training dataset (n = 377)				Validation dataset (n = 161)				SKC subgroup (n = 94)	
	Control (n = 240)		Keratoconus (n = 137)		Control (n = 90)		Keratoconus (n = 71)			
	OD	OS	OD	OS	OD	OS	OD	OS	OD	OS
Age [years]	28.13 ± 7.27		24.84 ± 6.55		27.63 ± 6.71		26.13 ± 4.97		24.68 ± 5.83	
Gender [n/%]										
Male	94 (39.0%)		97 (70.8%)		34 (37.8%)		50 (69.6%)		63 (67.0%)	
Female	147 (61.0%)		40 (29.2%)		56 (62.2%)		21 (30.4%)		31 (33.0%)	
KSS grade of the worse eye [n]										
KSS 0	236	238	6	6	88	88	4	4	1	1
KSS 1	2	1	67	50	1	1	30	22	55	47
KSS 2	2	1	33	40	1	1	9	17	38	46
KSS 3	—	—	15	24	—	—	18	19	—	—
KSS 4	—	—	14	9	—	—	7	5	—	—
KSS 5	—	—	2	8	—	—	3	4	—	—

Data are presented as mean ± SD, or number and percentages.

SKC, subclinical keratoconus; OD, right eye; OS, left eye; KSS, keratoconus severity score.

### Interocular consistency test

The distribution of corneal biomechanical properties is summarized in Table 3. The interocular consistency was poor in both the control and KC groups in parameters such as HCT, A1DeflAmp and A2DeflAmp (Table 3). However, in general, the left and right eyes in the control group had good consistency, which was better than that of the patients with keratoconus. In the parameters such as A1V, A2V, DAR2, DAR1, ARTh, SSI and IR, the interocular consistency in the KC group was extremely poor (Table 3).

**TABLE 3:** Interocular consistency test of corneal biomechanical parameters in the control and keratoconus groups.

	Control (n = 331)				Keratoconus (n = 207)			
Biomechanical parameters	OD	OS	ICC^a^	p for ICC	Better eye	Worse eye	ICC^a^	p for ICC
Pachy [μm]	556.91 ± 30.98	557.7 ± 31.75	0.956	<0.001	506.47 ± 42.85	475.24 ± 38.46	0.588	<0.001
A1T [ms]	7.38 ± 0.29	7.40 ± 0.28	0.857	<0.001	7.29 ± 0.26	7.16 ± 0.26	0.720	<0.001
A1V [m/s]	0.15 ± 0.02	0.15 ± 0.02	0.725	<0.001	0.16 ± 0.02	0.17 ± 0.02	0.421	<0.001
A2T [ms]	21.51 ± 0.46	21.52 ± 0.46	0.844	<0.001	22.20 ± 0.55	22.33 ± 0.56	0.846	<0.001
A2V [m/s]	−0.28 ± 0.03	−0.28 ± 0.03	0.621	<0.001	−0.29 ± 0.04	−0.31 ± 0.05	0.388	<0.001
HCT [ms]	16.84 ± 0.57	16.91 ± 0.51	0.365	<0.001	17.17 ± 0.54	17.16 ± 0.52	0.260	<0.001
PD [mm]	5.33 ± 0.27	5.31 ± 0.26	0.770	<0.001	5.37 ± 0.26	5.39 ± 0.25	0.727	<0.001
A1DeflAmp [mm]	0.09 ± 0.01	0.09 ± 0.01	0.258	<0.001	0.09 ± 0.01	0.10 ± 0.01	0.295	<0.001
HCDeflAmp [mm]	0.95 ± 0.10	0.94 ± 0.10	0.810	<0.001	1.02 ± 0.11	1.10 ± 0.13	0.579	<0.001
A2DeflAmp [mm]	0.10 ± 0.01	0.10 ± 0.01	0.369	<0.001	0.10 ± 0.01	0.11 ± 0.02	0.211	0.001
A1DeflArea [mm^2^]	0.17 ± 0.03	0.19 ± 0.04	0.164	0.001	0.17 ± 0.03	0.19 ± 0.03	0.122	0.039
HCDeflArea [mm^2^]	3.61 ± 0.52	3.57 ± 0.51	0.779	<0.001	3.81 ± 0.56	4.04 ± 0.60	0.671	<0.001
A2DeflArea [mm^2^]	0.22 ± 0.04	0.22 ± 0.04	0.256	<0.001	0.22 ± 0.05	0.24 ± 0.06	0.233	<0.001
A1ΔArcL [mm]	−0.02 ± 0.00	−0.02 ± 0.00	0.433	<0.001	−0.02 ± 0.00	−0.02 ± 0.01	0.319	<0.001
HCΔArcL [mm]	−0.12 ± 0.02	−0.12 ± 0.02	0.647	<0.001	−0.11 ± 0.03	−0.11 ± 0.03	0.499	<0.001
A2ΔArcL [mm]	−0.02 ± 0.00	−0.02 ± 0.00	0.498	<0.001	−0.02 ± 0.01	−0.02 ± 0.01	0.323	<0.001
MaxIR [mm^-1^]	0.17 ± 0.01	0.17 ± 0.01	0.583	<0.001	0.19 ± 0.02	0.22 ± 0.03	0.293	<0.001
DAR2	3.95 ± 0.35	3.94 ± 0.34	0.873	<0.001	4.62 ± 0.60	5.37 ± 0.90	0.294	<0.001
DAR1	1.51 ± 0.04	1.50 ± 0.04	0.749	<0.001	1.57 ± 0.06	1.63 ± 0.07	0.394	<0.001
ARTh	608.71 ± 118.78	675.69 ± 140.06	0.781	<0.001	420.04 ± 150.92	244.59 ± 100.13	0.278	<0.001
bIOP [mmHg]	15.62 ± 2.16	15.74 ± 2.16	0.799	<0.001	14.63 ± 2.05	14.03 ± 2.19	0.655	<0.001
IR [mm^-1^]	7.98 ± 0.83	7.91 ± 0.89	0.798	<0.001	9.74 ± 1.53	11.64 ± 1.88	0.345	<0.001
SP-A1	117.61 ± 15.38	115.02 ± 16.35	0.782	<0.001	88.52 ± 20.72	67.22 ± 20.45	0.509	<0.001
SSI	0.85 ± 0.12	0.86 ± 0.13	0.770	<0.001	0.79 ± 0.14	0.70 ± 0.13	0.475	<0.001

Data are presented as mean ± SD.

OD, right eye; OS, left eye; better eye, eye with a lower KSS, grade; worse eye, eye with a greater KSS, grade; ICC, intraclass correlation coefficient.

^a^ ICC, was calculated using *Two-way random* model and *Consistency* type.

### Comparison of interocular asymmetry

**FIGURE 2:**
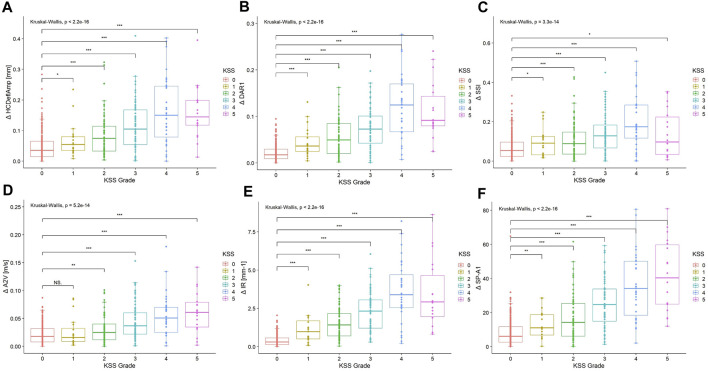
Boxplots of interocular asymmetry values of **(A)** Δ HCDeflAmp, **(B)** Δ DAR1, **(C)** Δ SSI, **(D)** Δ A2V, **(E)** Δ IR and **(F)** Δ SP-A1 for the control and keratoconus with different KSS grading of the worse keratoconic eye. The asterisks show the result of the Kruskal–Wallis test (NS, not significant; *, significant on a *p* < 0.05 level; **, significant on a *p* < 0.01 level; ***, significant on a *p* < 0.001 level). KSS, keratoconus severity score; Δ HCDeflAmp, asymmetry of the deflection amplitude of the corneal apex at the highest concavity; Δ DAR1, asymmetry of the ratio between the central deformation and the average of the peripheral deformation determined at 1.00 mm; Δ SSI, asymmetry of the stress‒strain index; Δ A2V, asymmetry of the speed of the corneal apex at the second applanation; Δ IR, asymmetry of the integrated radius; Δ SP-A1, asymmetry of the stiffness parameter at the first applanation.

The interocular asymmetry values of almost all analyzed variables were greater in the keratoconus group than in the control group, as listed in Table 4. In addition, the disagreement increased with the severity of the worse keratoconic eye in most parameters (Table 4). However, it is worth noting that in the analysis of interocular asymmetry of some descriptors, including Δ DAR1, Δ IR, Δ SSI, etc., reversals were observed in KSS grade 5 patients (Table 4; Figure 2).

**TABLE 4:** Interocular asymmetry values in the control and keratoconus of different severities.

Biomechanical parameters	Control (n = 331)	Keratoconus (n = 207)	p^a^	KSS of the worse eye in the keratoconus group					p^b^
				KSS 1 (n = 21)	KSS 2 (n = 73)	KSS 3 (n = 64)	KSS 4 (n = 31)	KSS 5 (n = 18)	
Pachy [μm]	7.13 ± 6.06	37.42 ± 31	<0.001	26.1 ± 43.71	28.12 ± 25.4	31.69 ± 17.62	53.26 ± 26.19	81.44 ± 33.76	<0.001
A1T [ms]	0.11 ± 0.1	0.19 ± 0.15	<0.001	0.14 ± 0.1	0.16 ± 0.14	0.18 ± 0.15	0.27 ± 0.17	0.25 ± 0.13	<0.001
A1V [m/s]	0.01 ± 0.01	0.02 ± 0.02	<0.001	0.01 ± 0.01	0.01 ± 0.01	0.02 ± 0.01	0.03 ± 0.02	0.02 ± 0.02	<0.001
A2T [ms]	0.19 ± 0.17	0.27 ± 0.2	<0.001	0.23 ± 0.17	0.25 ± 0.18	0.26 ± 0.2	0.36 ± 0.24	0.27 ± 0.22	<0.001
A2V [m/s]	0.02 ± 0.02	0.04 ± 0.03	<0.001	0.03 ± 0.03	0.03 ± 0.02	0.04 ± 0.03	0.05 ± 0.04	0.06 ± 0.04	<0.001
HCT [ms]	0.48 ± 0.39	0.5 ± 0.41	0.604	0.63 ± 0.43	0.49 ± 0.43	0.38 ± 0.29	0.61 ± 0.45	0.59 ± 0.51	0.128
PD [mm]	0.13 ± 0.12	0.15 ± 0.11	0.021	0.15 ± 0.14	0.16 ± 0.13	0.14 ± 0.1	0.16 ± 0.12	0.15 ± 0.09	0.293
A1DeflAmp [mm]	0.01 ± 0.01	0.01 ± 0.01	<0.001	0.01 ± 0	0.01 ± 0.01	0.01 ± 0.01	0.02 ± 0.01	0.03 ± 0.02	<0.001
HCDeflAmp [mm]	0.05 ± 0.04	0.11 ± 0.09	<0.001	0.06 ± 0.05	0.08 ± 0.07	0.12 ± 0.08	0.16 ± 0.11	0.16 ± 0.09	<0.001
A2DeflAmp [mm]	0.01 ± 0.01	0.02 ± 0.02	<0.001	0.01 ± 0.02	0.01 ± 0.01	0.02 ± 0.01	0.03 ± 0.02	0.04 ± 0.02	<0.001
A1DeflArea [mm^2^]	0.03 ± 0.04	0.03 ± 0.03	0.001	0.02 ± 0.02	0.03 ± 0.02	0.03 ± 0.02	0.05 ± 0.03	0.06 ± 0.05	<0.001
HCDeflArea [mm^2^]	0.26 ± 0.23	0.41 ± 0.33	<0.001	0.3 ± 0.3	0.37 ± 0.3	0.4 ± 0.32	0.55 ± 0.42	0.42 ± 0.27	<0.001
A2DeflArea [mm^2^]	0.04 ± 0.03	0.05 ± 0.05	0.001	0.05 ± 0.07	0.04 ± 0.04	0.05 ± 0.04	0.07 ± 0.06	0.11 ± 0.09	<0.001
A1ΔArcL [mm]	0 ± 0	0 ± 0	<0.001	0 ± 0	0 ± 0	0 ± 0	0.01 ± 0	0.01 ± 0.01	<0.001
HCΔArcL [mm]	0.01 ± 0.01	0.02 ± 0.02	<0.001	0.02 ± 0.03	0.02 ± 0.02	0.02 ± 0.02	0.03 ± 0.03	0.04 ± 0.03	<0.001
A2ΔArcL [mm]	0 ± 0	0.01 ± 0.01	<0.001	0 ± 0	0.01 ± 0	0.01 ± 0	0.01 ± 0.01	0.02 ± 0.01	<0.001
MaxIR [mm^-1^]	0.01 ± 0.01	0.04 ± 0.03	<0.001	0.02 ± 0.02	0.03 ± 0.02	0.04 ± 0.02	0.05 ± 0.03	0.05 ± 0.03	<0.001
DAR2	0.13 ± 0.11	0.92 ± 0.74	<0.001	0.49 ± 0.49	0.61 ± 0.48	0.94 ± 0.52	1.54 ± 1	1.59 ± 0.84	<0.001
DAR1	0.02 ± 0.02	0.07 ± 0.06	<0.001	0.05 ± 0.03	0.05 ± 0.04	0.07 ± 0.05	0.12 ± 0.07	0.11 ± 0.06	<0.001
ARTh	82.57 ± 71.51	192.14 ± 131.32	<0.001	125.74 ± 90.59	182.06 ± 120.45	192.18 ± 114.31	230.17 ± 156.36	244.8 ± 186.62	<0.001
bIOP [mmHg]	1.04 ± 0.91	1.39 ± 1.25	0.009	1.17 ± 0.92	1.3 ± 1.15	1.31 ± 1.2	1.91 ± 1.69	1.39 ± 1.13	0.076
IR [mm^−1^]	0.41 ± 0.37	2.21 ± 1.62	<0.001	1.15 ± 0.9	1.57 ± 1	2.28 ± 1.32	3.57 ± 2.03	3.44 ± 2.18	<0.001
SP-A1	8.03 ± 7.19	23.9 ± 17.49	<0.001	12.87 ± 8.11	16.65 ± 13.45	25.01 ± 14.44	35.74 ± 19.43	41.78 ± 21.2	<0.001
SSI	0.07 ± 0.06	0.13 ± 0.11	<0.001	0.09 ± 0.07	0.11 ± 0.09	0.14 ± 0.1	0.2 ± 0.14	0.14 ± 0.12	<0.001

Data are presented as mean ± SD.

KSS, keratoconus severity score.

The interocular asymmetry value, the absolute value of the right eye minus that of the left eye.

^a^ p for Mann‒Whitney *U* test between the control and keratoconus groups.

^b^ p for Kruskal‒Wallis test among the keratoconus patients with different severities.

### Diagnostic performance of interocular asymmetry

Among the interocular asymmetry values, Δ DAR2 and Δ IR achieve an AUC of over 0.9, while the others have a mediocre performance in distinguishing KC and SKC from controls (Table 5).

**TABLE 5:** Diagnostic performance of interocular asymmetry values and combined models for distinguishing the control from KC or SKC.

	Control vs. KC (training dataset)				Control vs. KC (validation dataset)				Control vs. SKC subgroup			
Interocular asymmetry parameters	Cutoff	AUC	Sensitivity	Specificity	Cutoff	AUC	Sensitivity	Specificity	Cutoff	AUC	Sensitivity	Specificity
Δ Pachy [μm]	16.5	0.875	0.730	0.958	17.5	0.878	0.746	0.922	16.5	0.786	0.591	0.939
Δ A1T [ms]	0.150	0.652	0.533	0.729	0.190	0.636	0.394	0.878	0.194	0.580	0.290	0.873
Δ A1V [m/s]	0.016	0.714	0.489	0.871	0.016	0.745	0.549	0.911	0.016	0.621	0.376	0.882
Δ A2T [ms]	0.162	0.609	0.701	0.483	0.149	0.641	0.746	0.533	0.197	0.600	0.591	0.612
Δ A2V [m/s]	0.048	0.673	0.365	0.938	0.051	0.689	0.338	0.989	0.054	0.581	0.204	0.970
Δ HCT [ms]	0.236	0.531	0.737	0.362	0.766	0.490	0.254	0.911	0.215	0.528	0.796	0.285
Δ PD [mm]	0.126	0.576	0.518	0.637	0.141	0.517	0.507	0.633	0.126	0.551	0.516	0.615
Δ A1DeflAmp [mm]	0.013	0.676	0.401	0.967	0.013	0.741	0.408	0.978	0.013	0.568	0.194	0.970
Δ HCDeflAmp [mm]	0.068	0.761	0.620	0.808	0.050	0.731	0.761	0.611	0.053	0.680	0.613	0.676
Δ A2DeflAmp [mm]	0.016	0.683	0.460	0.854	0.020	0.600	0.324	0.911	0.005	0.547	0.742	0.358
Δ A1DeflArea [mm^2^]	0.046	0.572	0.263	0.867	0.042	0.599	0.366	0.844	0.024	0.505	0.462	0.573
Δ HCDeflArea [mm^2^]	0.430	0.646	0.431	0.858	0.102	0.602	0.803	0.356	0.266	0.603	0.581	0.606
Δ A2DeflArea [mm^2^]	0.064	0.577	0.307	0.8211	0.041	0.587	0.507	0.644	0.034	0.527	0.559	0.521
Δ A1ΔArcL [mm]	0.004	0.691	0.474	0.854	0.004	0.694	0.535	0.856	0.004	0.620	0.333	0.855
Δ HCΔArcL [mm]	0.014	0.650	0.650	0.625	0.026	0.676	0.451	0.856	0.013	0.640	0.656	0.582
Δ A2ΔArcL [mm]	0.005	0.644	0.431	0.838	0.007	0.643	0.366	0.933	0.005	0.615	0.441	0.739
Δ MaxIR [mm^-1^]	0.020	0.828	0.686	0.887	0.016	0.870	0.789	0.844	0.016	0.786	0.688	0.815
Δ DAR2	0.300	0.907	0.781	0.921	0.299	0.923	0.859	0.889	0.300	0.874	0.688	0.912
Δ DAR1	0.046	0.820	0.620	0.938	0.053	0.839	0.662	0.922	0.047	0.748	0.473	0.921
Δ ARTh	132.542	0.778	0.628	0.858	104.008	0.788	0.775	0.756	128.893	0.753	0.570	0.836
Δ bIOP [mmHg]	0.950	0.583	0.569	0.608	1.650	0.544	0.310	0.844	1.350	0.555	0.430	0.718
Δ IR [mm^-1^]	0.945	0.903	0.745	0.908	1.185	0.928	0.732	0.978	1.140	0.866	0.602	0.952
Δ SP-A1	13.474	0.804	0.679	0.808	14.270	0.812	0.676	0.867	14.044	0.702	0.505	0.833
Δ SSI	0.084	0.694	0.628	0.708	0.100	0.691	0.592	0.778	0.085	0.637	0.570	0.700
BBI	0.477	0.952	0.854	0.971	0.493	0.954	0.901	0.956	0.190	0.913	0.828	0.894
MBI	0.513	0.961	0.854	0.967	0.421	0.970	0.887	0.956	0.484	0.968	0.849	0.988
BaBI	0.401	0.998	0.978	0.992	0.579	0.995	0.986	0.956	0.163	0.996	0.978	0.958
CBI	0.147	0.935	0.856	0.945	0.194	0.954	0.887	0.967	0.112	0.925	0.828	0.967
TBI	0.720	0.962	0.837	0.994	0.857	0.965	0.859	1.000	0.318	0.965	0.880	0.973
PRFI	0.442	0.965	0.909	0.988	0.502	0.968	0.915	0.989	0.296	0.969	0.924	0.991

KC, keratoconus; SKC, subclinical keratoconus; AUC, area under the receiver operating characteristic curve.

The logistic regression, based on 3 training datasets, produced 3 corresponding formulas:

$$\text{BBI}=\mathrm{EXP}\left(\text{Beta}1\right)/\left(1+\mathrm{EXP}\left(\text{Beta}1\right)\right)$$

$$\text{MBI}=\mathrm{EXP}\left(\text{Beta}2\right)/\left(1+\mathrm{EXP}\left(\text{Beta}2\right)\right)$$

$$\text{BaBI}=\mathrm{EXP}\left(\text{Beta}3\right)/\left(1+\mathrm{EXP}\left(\text{Beta}3\right)\right)$$

where Beta1 = B1*Δ DAR2 + B2*Δ ARTh + B3*Δ IR + B4 *Δ Pachy + B5 and B1 = 3.873, B2 = 0.387, B3 = 1.624, B4 = 2.948, B5 = 1.734. Beta2 = B1* A1V + B2* A2V + B3* DAR1 + B4 * ARTh + B5* IR + B6* SP-A1 + B7* Age + B8 and B1 = −1.539, B2 = 0.616, B3 = 0.376, B4 = −0.864, B5 = 2.748, B6 = −2.232, B7 = −0.424, B8 = −0.445. Beta3 = B1* A1V + B2* A2V + B3* ARTh + B4 * IR + B5* SP-A1 +B6*Δ DAR1 + B7*Δ ARTh + B8*Δ IR + B9 and B1 = −2.767, B2 = 2.173, B3 = −5.458, B4 = 8.473, B5 = −3.565, B6 = 2.352, B7 = 3.851, B8 = 8.322, B9 = 2.689. The values of all constants used in the equation were highly significant (*p* < 0.01).

**FIGURE 3:**
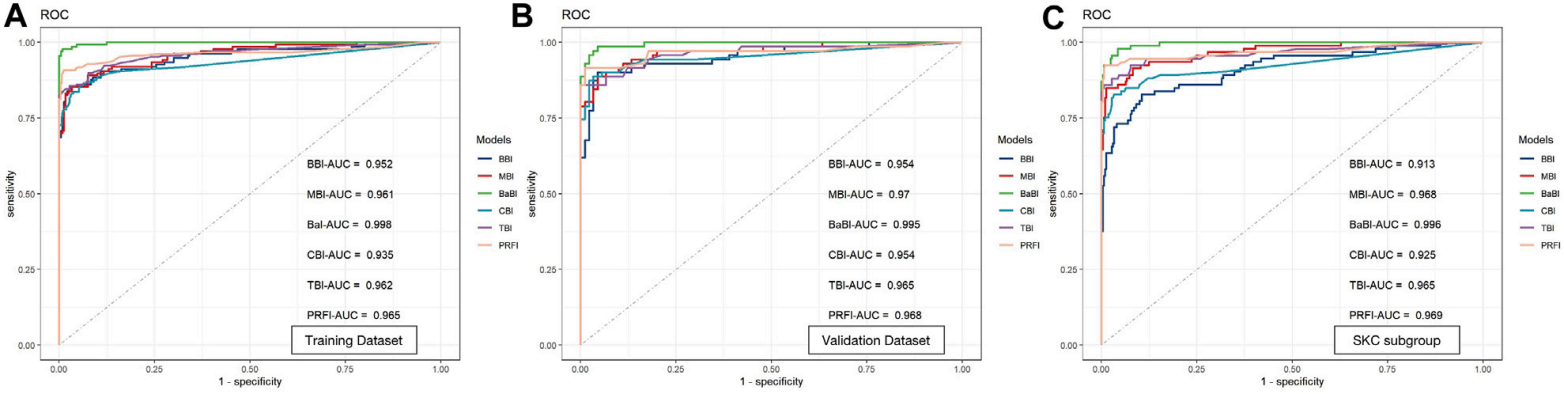
Receiver operating characteristic curves and area under the curve (AUC) of the newly established models and widely used monocular-based indices in **(A)** the training dataset, **(B)** the validation dataset and **(C)** the SKC subgroup. BBI, binocular biomechanical index; MBI, monocular biomechanical index; BaBI, binocular assisted biomechanical index; CBI, Corvis biomechanical index; TBI, tomographic and biomechanical index; PRFI, Pentacam random forest index.

The BBI performs much better than the individual interocular asymmetry values in the training dataset with an AUC of 0.952, and the MBI has an equivalent AUC of 0.961 (*p* = 0.613, Delong’s test). With a cutoff value of 0.401, the joint model BaBI achieved a higher AUC than the MBI (0.998 vs. 0.961, *p* < 0.001) and widely used monocular-based indices including CBI (0.998 vs. 0.935, *p* < 0.001), TBI (0.998 vs. 0.962, *p* < 0.001) and PRFI (0.998 vs. 0.965, *p* = 0.001) (Table 5; Figure 3). In addition, interocular asymmetry values have been proven to benefit monocular parameters in diagnosing KC (BaBI vs. MBI: NRI = 0.1505 ± 0.0646, *p* < 0.001, IDI = 0.2257 ± 0.0491, *p* < 0.001).

In the internal validation dataset, the BaBI also reached a higher AUC than the BBI (0.995 vs. 0.954, *p* = 0.017), MBI (0.995 vs. 0.970, *p* = 0.033), CBI (0.995 vs. 0.954, *p* = 0.022) and TBI (0.995 vs. 0.965, *p* = 0.048) (Table 5; Figure 2). In addition, the gain brought by the interocular asymmetry values to the monocular parameters was also considerable (BaBI vs. MBI: NRI = 0.1401 ± 0.0944, *p* = 0.004, IDI = 0.1779 ± 0.0721, *p* < 0.001).

For distinguishing the SKC and normal individuals, the combined model BaBI reached the highest AUC of 0.996 with a cutoff value of 0.163 and maintained an advantage over the BBI (0.996 vs. 0.913, *p* < 0.001) and the monocular models including MBI (AUC 0.996 vs. 0.968, *p* = 0.006), CBI (0.996 vs. 0.935, *p* < 0.001) and TBI (0.996 vs. 0.965, *p* = 0.030) (Table 5; Figure 2). The sensitivity of the BaBI model is 97.8%, which is higher than those of the monocular models (Table 5). In the analysis of NRI and IDI, the interocular asymmetry values also increased the monocular parameters (BaBI vs. MBI: NRI = 0.1301 ± 0.0653, *p* < 0.001, IDI = 0.1518 ± 0.0496, *p* < 0.001).

**FIGURE 4:**
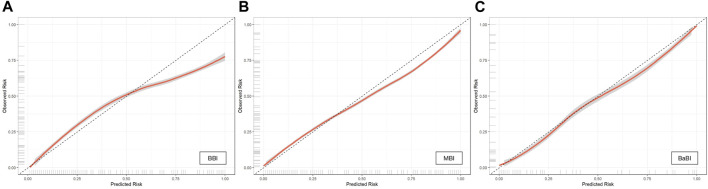
Calibration curves of **(A)** BBI, **(B)** MBI and **(C)** BaBI in the training dataset. BBI, binocular biomechanical index; MBI, monocular biomechanical index; BaBI, binocular assisted biomechanical index.

**FIGURE 5:**
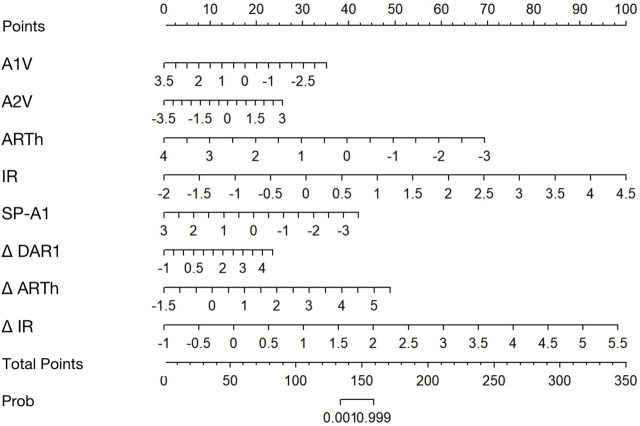
Nomogram for predicting KC from controls using the binocular-assisted biomechanical index (BaBI). A1V, speed of the corneal apex at the first applanation; A2V, speed of the corneal apex at the second applanation; ARTh, Ambrósio relational thickness to the horizontal profile; IR, integrated radius; SP-A1, stiffness parameter at the first applanation; Δ DAR1, asymmetry of the ratio between the central deformation and the average of the peripheral deformation determined at 1.00 mm; Δ ARTh, asymmetry of the Ambrósio relational thickness to the horizontal profile; Δ IR, asymmetry of the integrated radius.

The calibration curves of all established models were all good, but among them, the combined BaBI model showed the best overall discrimination (Figure 4). The nomogram of BaBI was drawn to provide a visualized convenient tool for clinical practice (Figure 5).

## Discussion

Although AI diagnostic models for distinguishing KC and SKC based on morphological parameters or images have been developed conspicuously over the decade, models based on biomechanical parameters are conversely rare (Vinciguerra et al., 2016; Steinberg et al., 2017). To improve the diagnostic ability for KC and especially SKC, the emphasis should be placed on utilizing biomechanical properties, which represent more intrinsic and subtle changes than morphology. In view of the asymmetric clinical manifestations of keratoconus, this study analyzed and compared the interocular asymmetry in biomechanical properties between normal subjects and patients with keratoconus and evaluated the potential to establish diagnostic models for KC and SKC based on this interocular feature.

We found that biomechanical parameters generally exhibit good interocular consistency in the eyes of normal individuals. However, the consistency is reduced and the interocular asymmetry values increase in keratoconic eyes, especially in parameters including A1V, A2V, HCT, MaxIR, DAR2 and DAR1, etc. Eppig et al. (2018) analyzed biomechanical parameters measured by the Ocular Response Analyzer (Reichert Inc., Depew, United States) and drew the same conclusion that interocular asymmetry is larger in keratoconus than in normal eyes. In contrast to the biomechanical parameters, the biomechanically corrected intraocular pressure (bIOP) has good interocular consistency in patients with KC. We suppose this suggests that although intraocular pressure affects the measurement of biomechanical parameters, it should not be the initial factor that causes the biomechanical changes in KC.

Eppig and others also reported that the interocular asymmetry of corneal hysteresis (CH) and corneal resistance factor (CRF) increased with the severity of KC, but reversal occurred in the severest patients classified as TKC stage 4 (Eppig et al., 2018). In this study, not only were similar close correlations of interocular asymmetry and severity observed, but the similar inverse behavior in severe keratoconus was also found. The inverse might be attributed to the relatively small sample in KSS grade 5. However, this does not affect the promising prospect of applying interocular asymmetry in the diagnosis of SKC.

It is worth noting that corneal biomechanical properties are influenced by some factors. Considerable corneal sclerosis and reduction of corneal viscoelastic properties with age were observed, which could be affected by age-related nonenzymatic cross-linking (Kotecha et al., 2006; Elsheikh et al., 2007; Matalia et al., 2016). The stress‒strain behavior of biological tissue is nonlinear, which means that the stress and strain of the cornea and sclera increase with intraocular pressure, causing a rise in the tangent modulus and influencing immediate corneal stiffness (Ethier et al., 2004; Eliasy et al., 2019). In addition, bIOP is an integral component of other Corvis ST parameters, such as SP-A1. Thus, bIOP also has an impact on the calculation of the classic biomechanical diagnostic parameter CBI (Vinciguerra et al., 2016; Roberts et al., 2017). Corneal thickness may cause differences in biomechanical parameters as well, with thicker corneas having greater dampening properties (Kotecha et al., 2006). To eliminate the influence of these factors, they were all included in the modeling in this study.

Individual interocular asymmetry values perform poorly in diagnosing keratoconus, while combined models perform well. Xian et al. (2023) reported that the AUC of the individual interocular asymmetry values did not exceed 0.9, but the logistic model combining ΔDAR2, ΔIR, and age reached a high AUC of 0.922 in identifying keratoconus. Similar results were achieved in the present study. Although inspiring outcomes were observed, we still have an objective view that the significance of interocular asymmetry lies in assisting monocular model diagnosis rather than replacing it. Therefore, an additional monocular parameter model, MBI, was established, and the classic monocular-based models were adopted as references. When comparing the interocular asymmetry model with the monocular models, no significant advantage was observed. However, the joint model that combined interocular asymmetry values and monocular descriptors had a better AUC than the monocular models, including the MBI, CBI, TBI, and PRFI. The NRI and IDI analysis proved that the interocular asymmetry values indeed bring gain to the monocular parameters in KC diagnosis. Furthermore, we attempted to evaluate the possibility of applying the interocular features in SKC diagnosis.

The difficulty lies in identifying the true population with binocular subclinical keratoconus, since the most popular criterion for SKC is the normal eye with a confirmed keratoconic fellow eye (Henriquez et al., 2020). However, the progression of the keratoconic fellow eye in this population may far exceed that of SKC to the point where it does not meet the diagnosis of bilateral SKC. It is difficult to verify the auxiliary diagnostic value of the biomechanical interocular asymmetry values without a properly allocated SKC group. Due to limited research on interocular asymmetry, there are few reference criteria for grouping binocular SKC. Naderan and others defined the KCS group as consisting of patients suspected of bilateral KC (60 < KISA% < 100 in both eyes) or a combination of KCS and normal eyes (60 < KISA% < 100 in one eye and KISA% < 60 in the other eye) and revealed that the intereye asymmetry of anterior corneal astigmatism had the highest accuracy of 0.923 (Naderan et al., 2017). Henriquez et al. (2015) classified KC patients with binocular Kmax equal to or less than 48 D as the VEKC group and constructed a logistic regression model using the asymmetry of morphological descriptors that reached an AUC value of 0.9957. We ultimately referred to Galleti and others’ research and designated keratoconic patients with KSS grades of 0–2 as the SKC subgroup for model validation alone (Ruiseñor Vázquez et al., 2014; Galletti et al., 2015a).

In distinguishing SKC from normal individuals, BaBI maintained its advantage over the monocular-based models. The additive benefit of interocular asymmetry values to the monocular parameters was also remarkable. Due to the insignificant clinical changes in SKC, the performance of the monocular-based models applied to the SKC population is often worse than that in the KC population, with relatively low sensitivity (Ambrósio et al., 2017; Lopes et al., 2018). However, the sensitivity of the BaBI is apparently higher than that of all other models, which means that the joint model can contribute to reducing the false-negative rate and improving the screening ability of SKC before the refractive surgery.

Although the Corvis ST is currently a widely used corneal biomechanical measurement device, its evaluation of *in vivo* biomechanics is not perfect. Corvis ST measures tissue responses to global deformation forces; therefore, it is not possible to assess regional differences in biomechanical properties. In addition, air puff results in not only corneal displacement but also motion of the entire ocular tissue and aqueous fluid (Boszczyk et al., 2017; Maczynska et al., 2019), which makes it difficult to detect minute variations in spatial stiffness in cases of SKC where local weakness occurs (Pahuja et al., 2016). However, emerging biomechanical measurements might be a promising supplement to solve this challenge. Brillouin microscopy does not require any stimulation or corneal deformation but relies on optical resolution. Shao et al. reported a distinction between early-stage KC and normal groups in the analysis of regional differences between cone and outside-cone regions and greater interocular differences of Brillouin shifts in stage-I KC patients than in normal controls (Shao et al., 2019). Optical coherence elastography (OCE) has high dynamic spatial and temporal resolution and can achieve flexible submicron stimulation using micro air pulses (Lan et al., 2021). OCE is expected to be able to image small amplitude and high-speed processes related to the propagation of elastic waves in the local cornea, thereby further evaluating the regional distribution characteristics of biomechanical interocular features (e.g., mirror symmetry) and providing a theoretical basis for assisting in the diagnosis of SKC.

There are limitations in this study. We relied on only one high-quality examination per eye to conduct the statistical analysis. There may be concerns about the repeatability of biomechanical measurements, but studies have shown that most biomechanical parameters have good stability in repeated measurements (Herber et al., 2020; Serbecic et al., 2020; Wang et al., 2021). Second, this is a single-center study with a relatively small sample size and a lack of ethnic diversity. We hypothesize that calculating the interocular asymmetry may help reduce the bias caused by the system in comparison to monocular descriptors. Despite this, the normal range of biomechanical interocular asymmetry values proposed in this article and the diagnostic ability of newly constructed models need further clinical testing in different regions and races. Thirdly, this study lacks long-term follow-up and longitudinal data. Considering that secondary corneal ectasia may occur within more than 10 years after refractive surgery, the 2-year follow-up of the control group is relatively short. The limited follow-up time and lack of longitudinal data in the keratoconus group also limit the application of the conclusions obtained in this study—biomechanical interocular asymmetry values increase with the severity of keratoconus—in the assessment of keratoconus progression. After obtaining longer-term follow-up and longitudinal data, the conclusions of this study can be further validated and improved.

In conclusion, there is currently a lack of research on biomechanical interocular asymmetry. In this study, we observed that the biomechanical interocular consistency in patients with keratoconus was lower than that in normal people and clarified the normal scope of interocular asymmetry values of biomechanical descriptors. We found that most interocular biomechanical asymmetry values increase along with the severity of the disease, but the inverse exists in a minority of descriptors in severe KC grading KSS 5. The interocular asymmetry of corneal biomechanical properties performs well in the diagnosis of KC and SKC and has an exact auxiliary value for monocular parameters.

## Data Availability

The original data presented in the study are included in the article/Supplementary Material, further inquiries can be directed to the corresponding author.
